# Emma J. Brophy

**Published:** 1899-03

**Authors:** 


### OBITUARY.

### Emma J. Brophy.

Died February 6th, 1899, at 176 Ashland Avenue, Chicago, Ills., Emma J. Brophy, beloved wife of Dr. T. W. Brophy.

This great affliction which has come upon Dr. Brophy is one that will produce a sad impression upon the entire profession, and especially upon those who know Dr. Brophy and his beloved wife.

Mrs. Brophy possessed in a large measure the benignant qualities of womanhood. Her life was one of broadest beneficence, reaching out and influencing all with whom she came in contact, and especially was her kindness manifested to all those who were in need and trouble. She was never happier than when doing some kindly service for others. Her home was a lovely one to which she added the brightness of her inestimable life. She was one of those for whom the world was better because she was in it, and her removal will cause a void in many respects that will not be easily filled.

The sympathy of all the friends and acquaintances of Dr. Brophy will go out to him in full measure because of this bereavement. The remembrance of the fact of her life and character will tend to mitigate the weight of sorrow which has come upon the Doctor. May he be comforted and strengthened and helped to bear this great calamity.

